# The Elongator Subunit Elp3 Regulates Development, Stress Tolerance, Cell Cycle, and Virulence in the Entomopathogenic Fungus *Beauveria bassiana*

**DOI:** 10.3390/jof8080834

**Published:** 2022-08-10

**Authors:** Qing Cai, Juanjuan Wang, Jiatao Xie, Daohong Jiang, Nemat O. Keyhani

**Affiliations:** 1 College of Plant Science and Technology, Huazhong Agricultural University, Wuhan 430070, China; 2 Department of Microbiology and Cell Science, University of Florida, Gainesville, FL 32611, USA; 3 Department of Biotechnology, School of Biological Science and Biotechnology, University of Jinan, Jinan 250022, China

**Keywords:** histone acetyltransferase, gene transcription, cell cycle, asexual development, autophagy, multidrug transporter

## Abstract

Transcriptional activity is mediated by chromatin remodeling, which in turn is affected by post-translational modifications, including histone acetylation. Histone acetyltransferases (HATs) are capable of promoting euchromatin formation and then activating gene transcription. Here, we characterize the Elp3 GNAT family HAT, which is also a subunit of Elongator complex, in the environmentally and economically important fungal insect pathogen, *Beauveria bassiana.* BbElp3 showed high localization levels to mitochondria, with some nuclear and cytoplasmic localization also apparent. Targeted gene knockout of *BbElp3* resulted in impaired asexual development and morphogenesis, reduced tolerances to multiple stress conditions, reduced the ability of the fungus to utilize various carbon/nitrogen sources, increased susceptibility to rapamycin, and attenuated virulence in bioassays using the greater wax moth, *Galleria mellonella*. The Δ*BbElp3* mutant also showed disrupted cell cycle, abnormal hyphal septation patterns, and enlarged autophagosomes in vegetative hyphae. Transcriptome analyses revealed differential expression of 775 genes (DEGs), including 336 downregulated and 438 upregulated genes in the Δ*BbElp3* strain as compared to the wild type. Downregulated genes were mainly enriched in pathways involved in DNA processing and transcription, cell cycle control, cellular transportation, cell defense, and virulence, including hydrophobins, cellular transporters (ABC and MFS multidrug transporters), and insect cuticular degrading enzymes, while upregulated genes were mainly enriched in carbohydrate metabolism and amino acid metabolism. These data indicate pleiotropic effects of BbElp3 in impacting specific cellular processes related to asexual development, cell cycle, autophagy, and virulence.

## 1. Introduction

Histone acetylation is an important epigenetic post-translational modification controlling chromatin remodeling and transcriptional activity by altering chromatin structure [1,2]. Histone acetylation/deacetylation is a reversible process that is controlled by the activities of a family of histone acetyltransferases (HATs), which catalyze the addition of acetyl groups to specific histone amino acid residues, while histone deacetylases (HDACs) catalyze the removal of histone acetylation marks [3,4]. Patterns of histone acetylation typically correlate with gene activation, whereas histone deacetylation results in DNA compaction and transcriptional repression [5,6,7]. Elongator protein 3 (Elp3), a member of the GNAT family histone acetyltransferases, is the catalytic subunit of the Elongator complex, a highly conserved multitasking protein complex responsible for transcriptional elongation in eukaryotic cells [8,9,10,11]. The Elongator complex consists of six subunits, including Elp1 through Elp6, with Elp3 being the HAT catalytic subunit [12]. Elongator participates in a large array of distinct cellular processes that includes histone/non-histone acetylation, DNA methylation, and tRNA modification [10,13,14,15,16]. Yeast (and mammalian) Elp3 is capable of acetylating histones H3/H4 in vitro, and the HAT activity of Elp3 has been proven essential for its in vivo functioning [9,10,17]. Moreover, Elp3 can also function as a tRNA modification enzyme, catalyzing the synthesis of the 5-methoxycarbonylmethyl (mcm^5^) and 5-carbamoylmethyl (ncm^5^) groups present on uridines at the wobble position in tRNA [14], and as a consequence, Elp3 mutant cells exhibited defective mcm^5^U modification and also translated insufficient mRNAs enriched with –AA ending codons [14,18].

In general, Elp3 functions as the catalytic subunit of Elongator complex and is required for the activation of gene transcription in eukaryotes, with a number of links to stress responses [11,17,19,20]. In *Saccharomyces cerevisiae* (Saccharomycetales, Saccharomycetes), loss of *Elp3* resulted in repression of genes involved in stress tolerance and impaired growth [17]. In *Arabidopsis*, Elp3 was found to regulate the transcriptional of activation of stress- and auxin-related genes; *Elp3* deleted strains exhibited decreased tolerance to abscisic acid as well as increased sensitivity to drought conditions [11,21]. In mammalian systems (as human cells), Elp3 has been shown to be required for the transcription of the heat-shock response gene *Hsp70*, and disruption of *Elp3* showed cell hypersensitivity to heat stress [20]. In *Toxoplasma gondii*, a human parasite capable of causing potentially life-threatening disease in newborns and the immunocompromised, TgElp3 has been shown to be localized to the outer mitochondrial membrane and is essential for (parasite) cell viability [22]. Similarly, in HeLa cells, Elp3 was also shown to be localized in mitochondria [23]. Aside from work in yeast and mammalian cells, the functions of Elp3 homologs in other fungi, particularly pathogenic filamentous fungi, have only minimally been investigated. In the fungal plant pathogen *Fusarium graminearum*, *FgElp3*-deleted stains exhibited impaired sexual and asexual growth, decreased tolerance to oxidative-stress, and attenuated virulence on the host [24]. Similarly, in the rice blast fungus, *Pyricularia oryzae*, Δ*PoElp3* strains were defective in vegetative growth, stress response, and pathogenicity and showed increased autophagy [25]. However, characterization of Elp3 in insect pathogenic fungi has yet to be reported.

The filamentous entomopathogen, *Beauveria* *bassiana* (Hypocreales, Sordariomycetes), has been commercialized world-wide as a pest control agent capable of targeting a broad range of insect hosts [26,27]. The fungus infects insect percutaneously, with conidial spores attaching and germinating on the surface of the insect cuticle [28,29]. Growing hyphae then penetrate into the host integument via the combination of mechanical pressure and secretion of cuticle degrading enzymes, after which the fungus undergoes a dimorphic transition to produce single-celled, freely floating hyphal bodies in the insect hemocoel [30,31,32]. After depletion of the host nutrient in the hemolymph, the fungus works its way outwards, ultimately sporulating on the insect cadaver to complete its lifecycle [33,34]. Several parameters have been recognized as critical to the insect biological control potential of *B. bassiana,* including: conidial production (including yield, viability, and virulence); tolerances to abiotic/biotic stresses, especially oxidative, osmotic, heat, and UV; and the production of different virulence-related factors [35,36]. Asexual growth conditions have been shown to impact both stress resistance and virulence, which are regulated by an array of factors involved in conidial production, cell cycle control, multi-stress responses, and carbon/nitrogen utilization that feed into both viability and the infection process [37]. In terms of basic transcriptional regulation/machinery and chromatin structures, thus far, four *B. bassiana* HATs, including two GNAT superfamily members (Gcn5 and Spt10), a MYST superfamily member (Mst2), and a P300/CBP family member (Rtt109), have previously been characterized to variously affect developmental and virulence pathways in the fungus [38,39,40,41]. Here, we sought to characterize the contributions of the *B. bassiana* Elp3 (BbElp3), a GNAT superfamily member, in gene transcription, conidiation, cell cycle control, metabolism, stress response, and virulence via characterization of a Δ*BbElp3* targeted gene-knockout mutant. Transcriptomic analyses were also conducted to investigate downstream gene targets of BbElp3 that may be involved in asexual development, cell cycle, and fungus virulence of *B. bassiana*.

## 2. Materials and Methods

### 2.1. Bioinformatic Analysis of Elp3 Homolog in B. bassiana

The *B. bassiana* Elp3 homolog was identified by using the amino acid sequence of *S. cerevisiae* Elp3 (NP_015239) to query the *B. bassiana ARSEF 2860* genomic database (NCBI accession: NZ_ADAH00000000) [42]. The resultant sequences were then aligned with other representative fungi identified in the NCBI database (http://blast.ncbi.nlm. nih.gov/, accessed on 15 September 2014). Conserved domains were predicted with the SMART program (http://smart.embl-heidelberg.de, accessed on 15 September 2014), and phylogenetic analysis were conducted using the MEGA7 software (http://www.megasoftware.net, accessed on 15 September 2014). The molecular weight and isoelectric point of BbElp3 were predicted using ExPASy-Compute pI/Mw tool (https://web.expasy.org/compute_pi/, accessed on 15 September 2014).

### 2.2. BbElp3 Subcellular Localization Examination

The coding region of *BbElp3* was cloned using the primers cElp3-F/R (Table S1) with *B. bassiana* wild-type cDNA as template, and the eGFP protein coding region (GenBank accession code: U55763) was fused to the BbElp3 C-terminus. The fusion cassette *BbElp3::eGFP* was then cloned into plasmid pAN52-*bar* (phosphinothricin-resistance), and the whole vector was transformed into *B. bassiana* wild-type strain via blastospore transformation [43]. Putative transformants were screened by resistance to phosphinothricin (200 μg/mL) and examined under confocal microscopy (LSCM, FLUOVIEW FV3000, OLYMPUS, Tokyo, Japan). For determination of cellular localization, conidia were inoculated in SDB (4% glucose, 1% peptone, and 1% yeast extract) at 25 °C for 3 days at 150 rpm. Hyphal cells were harvested and costained with mitochondria-specific dye, Miro-tracker Red (Sigma, Tokyo, Japan), and nuclei-specific dye, DAPI (Sigma), for 30 min at 25 °C and observed under confocal microscopy.

### 2.3. Generation of B. bassiana Elp3 Mutants

The gene-knockout strain of *BbElp3* (Δ*BbElp3*) was constructed by homologous recombination using the *bar* as the selection marker conferring resistance to phosphinothricin, and the complementary strain (Δ*BbElp3::BbElp3*) was constructed via ectopic integration of the full-length *BbElp3* sequence (4416 bp total) using the *sur* selection marker (chlorimuron ethyl resistance) as described [44]. The primers used in the study are listed in Table S1. Putative transformants were screened by phosphinothricin (200 μg mL^−1^) or chlorimuron ethyl (10 μg mL^−1^) for the knockout and complementary strains, respectively. Transformants were single spore purified and verified again by PCR and Southern blotting (Table S1).

### 2.4. Western Blotting for Histone H3 Acetylation Detection

All strains including wild type, Δ*BbElp3*, and Δ*BbElp3::BbElp3* were inoculated in SDB liquid medium and grown with aeration for 3 d at 26 °C; then, the total protein was extracted and subjected to Western blotting as described [44]. Protein concentrations were quantified using the BCA Protein Assay Kit (KeyGen, Nanjing, China). Protein levels of β-actin and histone H3 were detected by use of an anti-β-actin antibody (Cell Signaling Technology, catalog #8457) and an anti-histone H3 antibody (Abcam, catalog #ab1791), respectively. The bulk acetylation of H3 was determined with H3Ac (H3 acetylation) antibody (Merck Millipore, catalog #06-599). After primary antibody binding, washing, and secondary antibody incubation using goat anti-rabbit IgG antibody (Boster, Wuhan, China), subsequent bands were detected by chemiluminescence examination (Amersham Biosciences, Shanghai, China). All experiments above were repeated three times. The intensities of all blots were quantified using ImageJ software (https://imagej.nih.gov/ij/, accessed on 20 July 2017).

### 2.5. Investigation of Vegetative Growth and Conidial Properties

To assess vegetative growth, 1 μL of 1 × 10^6^ conidia/mL suspensions of indicated strains were spotted on the center of plates. Media included: (i) rich media, SDAY (Sabouraud dextrose agar supplemented with yeast extract); (ii) minimal CZA (Czapek-Dox agar) (3% sucrose, 0.3% NaNO_3_, 0.1% K_2_HPO_4_, 0.05% KCl, 0.05% MgSO_4_, and 0.001% FeSO_4_ plus 1.5% agar); and (iii) 16 different CZA-derived media. The CZA-derived media were prepared by replacing the carbon source (sucrose) found in CZA with either glucose, fructose, lactose, maltose, mannitol, trehalose, glycerol, or NaAc or by replacing the nitrogen source (NaNO_3_) found in CZA with either NH_4_Cl, NaNO_2_, or NH_4_NO_3_. Plates were incubated for 8 days at 25 °C, and the diameters of all colonies were measured.

To assess stress responses, 1 μL of 1 × 10^6^ conidia/mL suspensions were spotted on CZA plates (control) or CZA-modified plates. The CZA-modified plates included (i) osmotic stress-causing agents, namely 0.4 M NaCl or 0.4 M KCl; (ii) oxidative stress-causing agents, namely 2 mM H_2_O_2_ or 0.02 mM menadione; (iii) TOR signaling inhibitor, namely 1 μg mL^−1^ rapamycin; (iv) cell-wall-perturbing agents, namely 100 μg mL^−1^ SDS or 10 μg mL^−1^ Congo Red; (v) DNA-damage-causing agents, namely 10 mM hydroxyurea (HU) or 0.05% methyl methanesulfonate (MMS); (vi) and two fungicides, namely 10 μg mL^−1^ carbendazim (CBD) or 10 μg mL^−1^ iprodione metabolite (IPM). The diameter of each colony was measured after 8 d incubation at 25 °C. Relative growth inhibition (RGI) index = (*S*_c_–*S*_s_)/*S*_c_×100; *S*_c_ and *S*_s_ denote the areas of control and stressed colonies, respectively.

To assess conidial production, 100 μL of 1 × 10^7^ conidia/mL suspensions were spread on SDAY plates and incubated for 9 days at 25 °C (L: D = 12:12 h). From day 4 to day 9, three of the 5 mm plugs were taken daily from each plate, immersed in 1 mL of 0.02% Tween 80, and treated to mild ultrasonication. Total conidial numbers were then quantified by counting using a hemocytometer. In addition, samples from 4 d SDAY cultures were examined by scanning electronic microscopy (Nova Nano SEM 450, Thermo FEI, Czech Republic) to observe the sporophore structures using protocols as described [44]. Flow cytometry analyses of conidia (2 × 10^4^ conidia, harvested from 7 d SDAY plates) and blastospores were performed to quantify the size and density (complexity) of cells according to the FSc and SSc readings as previously described [44]. Conidial hydrophobicity was assessed in a modified aqueous-organic system described before [45]. Conidial viability, heat tolerances, and UV-B resistance of each strain were also examined on GM germinating plates (2% sucrose, 0.5% peptone, 0.02% Tween 80, plus 1.5% agar) or GM plates with additional stress: (i) wet heat stress at 45 °C or (ii) UV-B irradiation as described [44].

### 2.6. Bioassays Using Gellaria Mellonella and Investigation of Virulence-Related Events

For the insect bioassays, *G. mellonella* (Lepidoptera, Pyralidae) larvae were used to examine fungal virulence either after topical application or intra-hemocoel injection. Briefly, ~30 larvae were either (1) immersed for 10 s in 30 mL 1 × 10^7^ conidia/mL suspensions for topical application or (2) injected with 5 μL 1 × 10^5^ conidia/mL suspensions for intra-hemocoel assays. Larvae were maintained at 25 °C for 10 days and examined every 12 h for mortality. Each bioassay experiment was repeated three times. The mean lethal times to kill 50% of larvae (LT_50_) were estimated by Probit analyses of the time–mortality trends. After 4 d post death, fungal hyphal on dead larval surfaces were observed.

Total extracellular protease and Pr1 protease activities were quantified as described previously [46]. Briefly, 100 mL of 1×10^5^ conidia/mL suspensions were inoculated in CZB liquid medium (containing 0.3% bovine serum albumin) at and grown with aeration at 25 °C for 4 d. Cultures were centrifuged at 13,200× *g* for 1 min at 4 °C, and the cell pellet collected and dried to measure total biomass (mg mL^−1^), with the supernatant fraction used for measuring (extracellular) enzyme activities. For total extracellular protease activity, 100 μL of the supernatant was mixed with 100 μL of azocasein solution (5 mg mL^−1^) and incubated for 1 h at 37 °C, after which 400 μL of 10% (*w*/*v*) trichloroacetic acid was added to end the reaction. After centrifugation at 12 000× *g* for 5 min, the supernatant was transferred to 700 μL of 525 mM NaOH, and the optical density at 442 nm (OD_442_) was recorded using a spectrophotometer. To assess the Pr1 activity, 100 μL of the supernatant fraction was mixed with 50 μL of 1 mM substrate (succinyl-(alanine)_2_-proline- phenylalanine-p-nitroanilide; Sigma) and 850 μL of 15 mM Tris HCl buffer (pH 8.5). The mixture was incubated for 1 h at 28 °C, and then, 250 μL of 30% acetic acid was added to end the reaction. After 15 min on ice, the mixture was centrifuged at 12,500× *g* for 5 min at 4 °C, and the OD of the final supernatant examined at 442 nm. One unit of enzyme activity was defined as the enzyme amount required for an increase of each OD value by 0.01 after 1 h reaction and quantified as U/mg extract.

Unicellular blastospore production was quantified as follows: 50 mL aliquots of 10^6^ conidia/mL suspensions were inoculated with CZB and TPB (an amended CZB mimic to insect hemolymph by using 3% trehalose as sole carbon source and 0.5% peptone as sole nitrogen source) and grown with aeration at 25 °C for 3d. Aliquots from the growing cultures were examined microscopically and the amount of the blastospores quantified using a hemocytometer. Fungal cells were also collected by centrifugation and dried to measure the biomass (mg/mL) as above. All phenotypic data were applied to one-way ANOVA analysis and Tukey’s honestly significant difference (HSD) test. All experiments were performed with three technical replicates and the entire experiment repeated three times.

### 2.7. Examination of Cell Cycle, Hyphal Septation Pattern, and Autophagosome Formation

To investigate the cell cycle and hyphal septation, 50 mL of 1 × 10^6^ conidia/mL was grown in SDB with aeration for 3 d at 25 °C. Growing cells were then collected and stained with calcofluor white (Sigma) for 15 min. Cell length and width were measured under a fluorescent microscope with ~50 hyphal cells of each strain using ImageJ software. For cell cycle analyses, 50 mL of 1 × 10^6^ conidia/mL were grown in NLB (4% glucose, 0.4% NH_4_NO_3_, 0.3% KH_2_PO_4_, and 0.3% MgSO_4_) with aeration for 3 d at 25 °C. Blastospores harvested from the NLB cultures were stained with propidium iodide and then examined by flow cytometry to determine the G_0_/G_1_, G_2_/M, and S phases of cells as described previously [44]. Blastospore cell size and density were also quantified according to the FSc and SSc readings from the flow cytometry, respectively.

To examine formation of autophagosomes, 50 mL of 1 × 10^6^ conidia/mL was grown in SDB with aeration for 3 d at 25 °C. Growing cells were then collected and washed twice in sterile dH2O and resuspended in fresh SDB containing rapamycin (10 μg mL^−1^) for 12 h incubation at 25 °C. Hyphal cells were collected, treated, and examined under transmission electron microscope (Tecnai G2 spirit TEM, Thermo FEI, Czech Republic) to observe the formation of autophagosomes in each cell [44]. The numbers and diameters of autophagosomes were measured with ~30 hyphal cells of each strains using ImageJ software.

### 2.8. Transcriptomic Analysis

Three replicates cultures of the Δ*BbElp3* and *B. bassiana* wild-type strains (6 samples total) were grown for 4 d on cellophane overlaid SDAY plates, after which the fungal cells were collected and sent to Personal Biotechnology Co., Ltd. (Shanghai, China) for transcriptomic analyses. Total RNA was extracted using RNA Trizol (Sigma), and the mRNA fraction was isolated using magnetic oligo (dT) beads. mRNAs were fragmented by ionic disruption and then used as templates for first-strand cDNA synthesis using random hexamer primers. Second-strand cDNA synthesis was then conducted using a cDNA Synthesis Kit (Sigma). After purification, end-repair, and single adenine addition, cDNA libraries were constructed with end-labeled adaptors. All samples were then sequenced using an Illumina HiSeq platform. All clean tags were filtered and aligned with the *B. bassiana* genome [42]. Transcripts were regarded to be significantly regulated if log_2_ (Δ*BbElp3*/WT ratio) < −1 (downregulated) or > 1 (upregulated) and false-discovery rate (FDR) < 0.01. All the data were normalized as fragments per kilobase of exon per million fragments mapped (FPKM). All identified DEGs were functionally annotated with FunCat category classification (https://elbe.hki-jena.de/fungifun/, accessed on 16 July 2019). Furthermore, Kyoto Encyclopedia of Genes and Genomes (KEGG) analysis (http://www.genome.jp/kegg/, accessed on 16 July 2019) was performed to identify various KEGG pathways influenced at the significant level of *p* < 0.05. Pathogen–host interactions were also analyzed on PHI database (http://www.phi-base.org/, accessed on 16 July 2019). The transcriptomics data have been deposited to the Sequence Read Archive (SRA) on NCBI (https://www.ncbi.nlm.nih.gov/, accessed on 13 April 2021) with the dataset identifiers PRJNA721731.

## 3. Results

### 3.1. Bioinformatic Features and Subcellular Localization of B. bassiana Elp3 and Construction of Deletion and Complemented Strains

The Elp3 homolog in *B. bassiana* (NCBI accession code: EJP64095; tag locus: BBA_07100) was identified as being encoded by a nucleotide sequence of 1790 bp containing one intron. The BbElp3 protein consists of 576 amino acids (molecular mass: 65.01 kDa; isoelectric point: 8.54) and contained an ELP3 super-family domain (residues 55–567), as typically seen in Elp3 homologs, with high similarities with *S. cerevisiae* (80%) and *Aspergillus nidulans* (88%) (Figure S1A). Overall, BbElp3 shared 72–97% high sequence identity with homologs found in yeasts and other filamentous fungi (Figure S1B).

**Figure 1:**
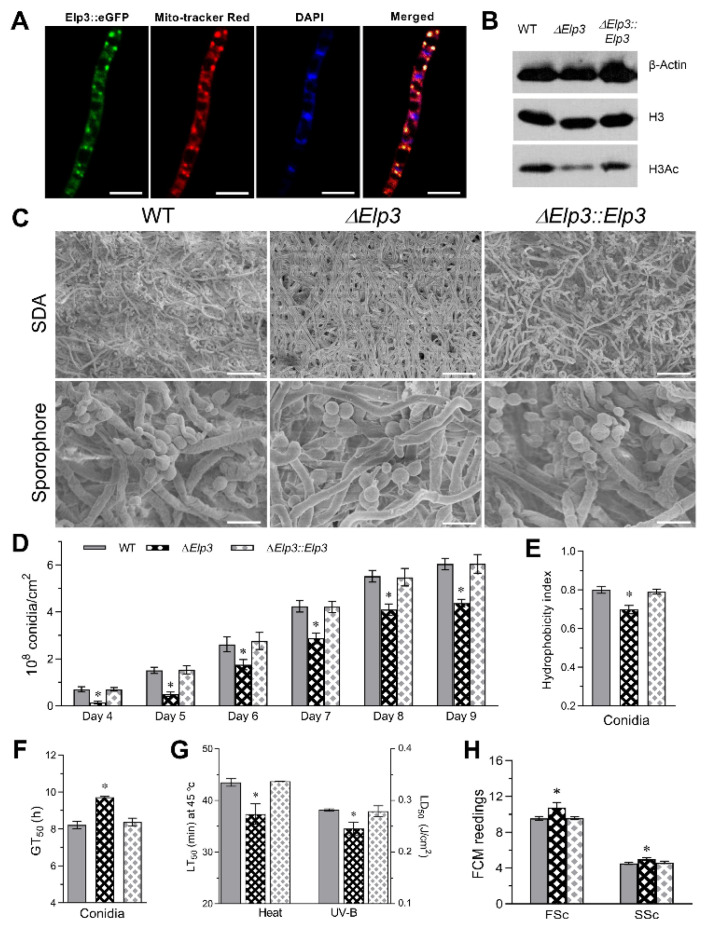
Characterization of *B. bassiana* Elp3: subcellular localization, histone acetyltransferase activity, and contribution to conidiation. (**A**) Representative images of cellular localization monitoring the BbElp3-GFP fusion protein. Scale bars: 10 µm. Hyphal cells were harvested from 3 d SDB cultures and costained with the mitochondria-specific dye (Mito-tracker Red) and nuclei-specific dye (DAPI). (**B**) Western blot analyses of histone H3 acetylation. (**C**) Scanning electron microscopy (SEM) images of aerial hyphae and sporophore of cells harvested from SDAY plats grown for 4 d. Scale bars: upper panels, 20 µm; lower panels, 5 µm. (**D**) Conidial yields assessed for each strain over a 9 d time course of growth on SDAY plates. (**E**) Conidial hydrophobicity indexes. (**F**,**G**) Conidial germination times (GT50) and conidial tolerances to heat stress and UV-B irradiation determined as detailed in the Methods section. (**H**) FACS analysis for cell size (FSc) and density (SSc) of aerial conidia grown for 7 d on SDAY. Asterisk indicates significant difference (*p* < 0.05) from unmarked (Tukey’s HSD). Error bars: SD from three technical replicates.

To investigate the subcellular localization of BbElp3, a transgenic strain expressing BbElp3::GFP fusion protein was cultivated in SDB for 3 days, and hyphal cells were costained with Mito-tracker Red and DAPI and then observed under microscope. Some diffuse eGFP signals could be seen throughout the cell with distinct punctate spots visible, similar to that seen after Mito-tracker Red staining (Figure 1A). Overall, these results showed strong overlap between the green fluorescence signal of BbElp3-eGFP with Mito-tracker Red staining of mitochondria, indicating the mitochondrial localization of BbElp3; however, cellular and nuclear localization of the protein was also noted.

In order to further probe the function of BbElp3, a targeted gene deletion (Δ*BbElp3*) and complemented (Δ*BbElp3::BbElp3*) strains were constructed as detailed in the Methods section. Transformants and putative knockout/complemented strains were screened by PCR and the correct integration events confirmed by Southern blotting analyses (Figure S2, primers used are listed in Table S1).

### 3.2. Role of BbElp3 in Histone H3 Acetylation, Sporulation, and Conidial Property

Western blotting using anti-β-Actin, anti-histone H3, and anti-histone H3ac antibodies as probes were applied to determine the contributions of BbElp3 in mediating acetylation of histone H3. As shown in Figure 1B, compared with the wild type, loss of *BbElp3* resulted in a significant (~50%, *p* < 0.01) decrease on H3 bulk acetylation levels as determined by densitometric quantification, indicating the conserved role of BbElp3 in histone H3 acetylation.

Scanning electronic microscopic (SEM) analyses of fungal cultures grown on SDAY plates initiated with conidial suspension revealed that in control strains (wild type and complemented), abundant conidiophores and conidia formed, whereas in contrast, similar rachises structures and conidia were sparser in Δ*BbElp3* cultures (Figure 1C). Consequently, the Δ*BbElp3* strain showed an ~68–80% decrease (*p* < 0.001) in conidial production on 4 and 5 d post inoculation, and lower (~26–33%, *p* < 0.01) conidial yields after 6–9 d growth (Figure 1C,D). The hydrophobicity index of 7-day-old conidia derived from the Δ*BbElp3* strain also showed a ~13% reduction (*p* < 0.001), indicating altered cell surface properties of the conidia produced by the Δ*BbElp3* strain (Figure 1E). Moreover, the time required for 50% of the conidia to germinate (GT_50_) was prolonged by ~18% (*p* < 0.001) for Δ*BbElp3* stain as compared to control conidia (Figure 1F).

Conidia derived from the Δ*BbElp3* also showed higher sensitivities to heat stress (~15%, *p* < 0.01) and UV irradiation (~15%, *p* < 0.01) (Figure 1G). In terms of cell morphology, fluorescence-activated cell sorter (FACS) analyses revealed a ~15% (*p* < 0.01) increase in the size of Δ*BbElp3* conidia versus wild type and a slight ~11% increase in conidial density, as indicated by the readings of forward scatter (FSc) and side scatter (SSc) detectors from the flow cytometry (Figure 1H).

### 3.3. Role of BbElp3 in Carbon/Nitrogen Metabolism and Multi-Stress Tolerance

To investigate the role of BbElp3 in carbon/nitrogen utilization, a range of media including SDAY, CZA, and CZA-derived media were applied to assess the growth of each strain. Compared with wild type, the vegetative growth of the Δ*BbElp3* mutant decreased by ~40% in rich media (SDAY, *p* < 0.001) and ~25% in minimal media CZA (*p* < 0.01, Figure S3). Furthermore, in CZA media amended with different carbon sources including six organic (glucose, fructose, lactose, maltose, mannitol, trehalose) and one inorganic carbon source (sodium acetate), growth was decreased by anywhere from ~20–50% for the Δ*BbElp3* mutant as compared to the control strains (*p* < 0.01). Similarly, in CZA media amended with different nitrogen sources including three inorganic nitrogen sources (NaNO_2_, NH_4_CL, NH_4_NO_3_), the growth of Δ*BbElp3* was also reduced by ~11–20% compared to the control strains (Figure S3).

**Figure 2:**
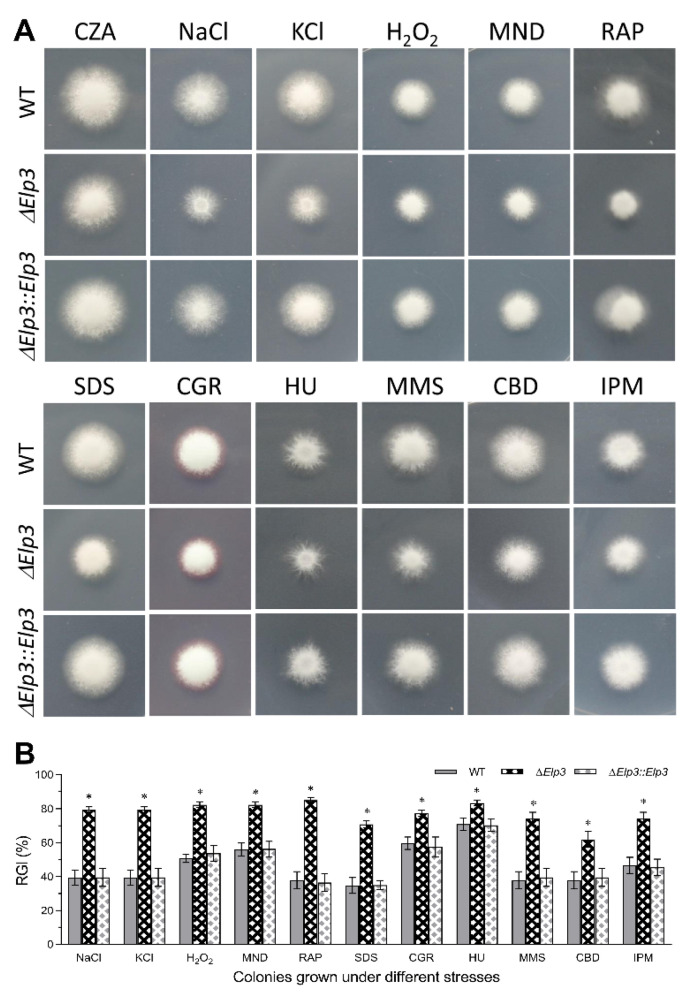
Contribution of BbElp3 to multi-stress tolerances. (**A**,**B**) Representative images and quantification of relative growth inhibition (RGI) of fungal colonies grown at 25 °C for 8 d on CZA supplemented with NaCl (0.4 M), KCl (0.4 M), H_2_O_2_ (2 mM), menadione (MND; 0.02 mM), rapamycin (RAP, 1 μg mL^−1^), SDS (100 μg mL^−1^), Congo Red (CGR; 10 μg mL^−1^), hydroxyurea (HU; 10 mM), methyl methanesulfonate (MMS, 0.05%), carbendazim (CBD, 10 μg mL^−1^), and iprodione metabolite (IPM, 10 μg mL^−1^). All colonies were initiated by spotting 1 μL of 1 × 10^6^ conidia/mL suspension on the plates. All experiments were performed three times, with each containing three technical replicate plates. Error bars = ± SD. Asterisk indicate significant difference (*p* < 0.05) from unmarked (Tukey’s HSD). Error bars: SD from three technical replicates.

In terms of stress responses, the Δ*BbElp3* strains showed reduced tolerances to various stress inducing agents, including those resulting in osmotic and oxidative stress, cell-wall-perturbing agents, DNA-damage-causing agent, and antibiotics (fungicides) (Figure 2). Deletion of *BbElp3* resulted in significantly decreased (~40%, *p* < 0.01) tolerances to salt (i.e., osmotic stress) induced by NaCl and KCl, with a smaller effect seen for sorbitol (~12%, *p* < 0.01, data not shown in graph). Similarly, a decrease (~25–25%, *p* < 0.01) in cellular tolerances to H_2_O_2_ and menadione as well as to the cell-wall-perturbing agents (~20–40%, *p* < 0.01) sodium dodecyl sulfate and Congo Red were noted. Increased (~12–36%, *p* < 0.01) sensitivity to hydroxyurea and methyl methanesulfonate and to carbendazim, iprodione, and rapamycin were noted for the Δ*BbElp3* strain as compared to the wild type and complemented strains (~25–30%, *p* < 0.01, Figure 2).

### 3.4. Influence of BbElp3 Deletion on Cell Cycle Progression and Hyphal Septation

**Figure 3:**
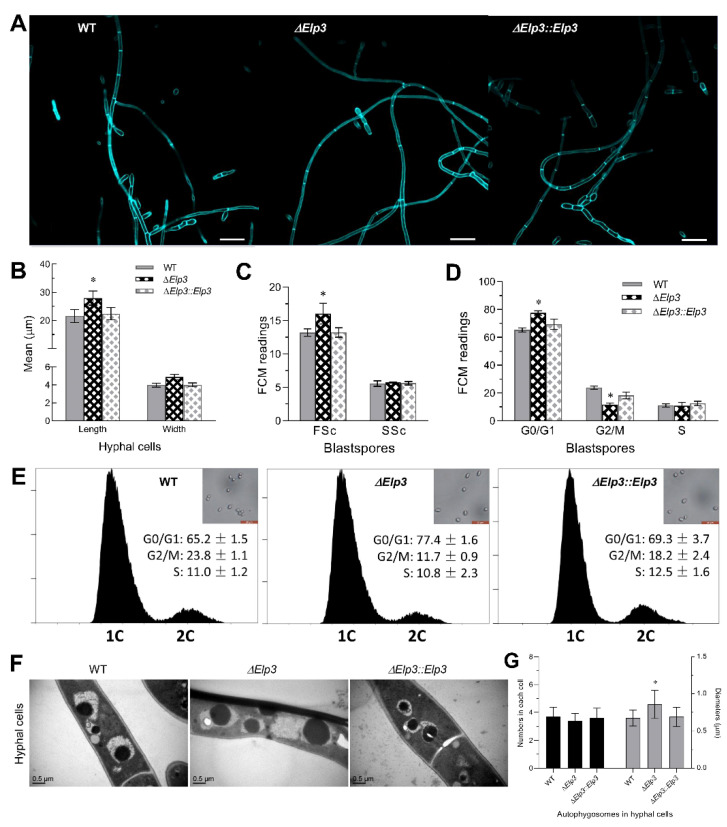
Impact of loss of *Elp3* on cell cycle, hyphal septation, and autophagosome formation in *B. bassiana*. (**A**) Representative microscopic of hyphal cells (3 d SDB cultures) stained with calcofluor white. Scale bars = 20 µm. (**B**) Quantification of mean hyphal cell length and width. (**C**) FACS analyses of blastospore (harvested from NLB cultures grown for 3 d) cell size (FSc) and cell density (SSc). (**D**,**E**) FACS analysis for blastospore cell cycle progression. (**F**) Representative TEM images of hyphal cells (3 d SDB cultures) treated with 10 μg mL^−1^ rapamycin for 12 h. Scale bars = 0.5 µm. (**G**) Quantification of the average numbers and diameters of autophagosomes in each rapamycin-treated hyphal cell. Asterisk indicates significant difference (*p* < 0.05) from unmarked (Tukey’s HSD). Error bars: SD from three technical replicates.

To explore the role of BbElp3 in fungal cellular development, the pattern of hyphal septation during growth as examined in cells were collected after 72 h growth in SDB and stained with the cell-wall-specific dye calcofluor white (CFW, Figure 3A,B). Microscopic visualization of the cells indicated altered (fewer) septal bands seen in hyphae of the Δ*BbElp3* strain, and overall, the hyphae of the Δ*BbElp3* strain were longer (~30%, *p* < 0.01) than those of control strains, with no obvious increase in terms of cell width. To examine effects on the unicellular blastospore stage of *B. bassiana* growth, cells from the Δ*BbElp3* mutant and control strains were harvested after 3 d growth in NLB (nitrogen-limited broth, which promotes formation of blastospores). The resultant cells were stained with propidium iodide and analyzed by flow cytometry. Compared to the wild type, the blastospores derived from the Δ*BbElp3* mutant strain showed a slight increase (~16%) in size and overall cell density (~6%) (Figure 3C,D). However, the Δ*BbElp3*-derived cells showed significantly longer G_0_/G_1_ (~19%, *p* < 0.01) and shorter G_2_/M (~50%, *p* < 0.01) but were unaffected in S phase progression (Figure 3D,E), indicating a pronounced G_0_/G_1_ phase arrest. Furthermore, transmission electron microscopy revealed the formation of enlarged autophagosomes in Δ*BbElp3* cells harvested after 3 d growth in SDB in the presence of rapamycin as compared to controls. However, no obvious changes in terms of the average numbers of autophagosomes in each hyphal cell were seen between the mutant and wild-type parent under these conditions (Figure 3F,G).

### 3.5. BbElp3 Deletion Attenuates B. bassiana Virulence

**Figure 4:**
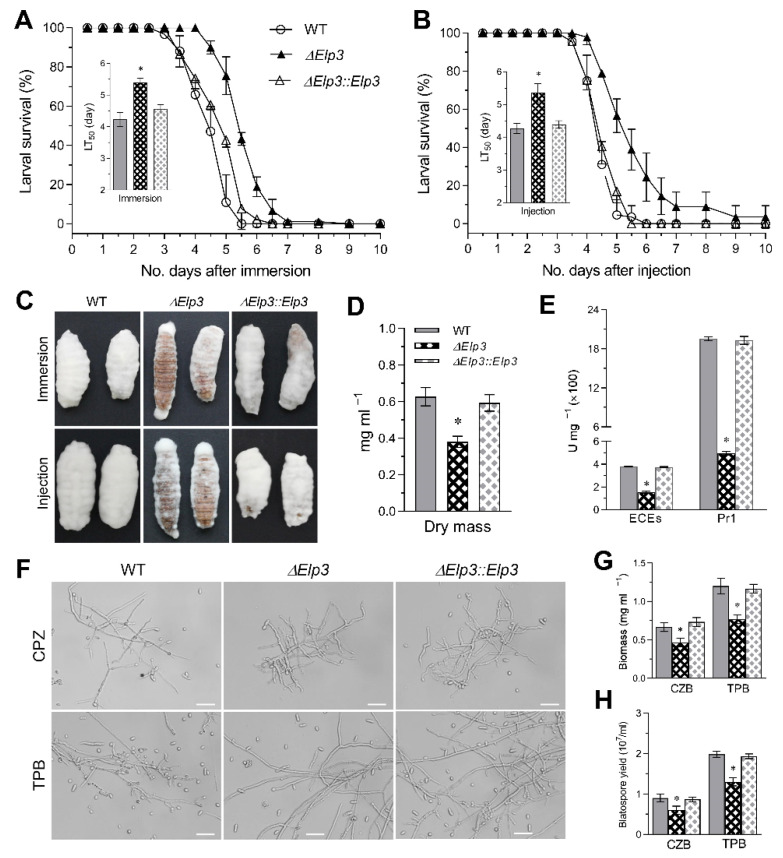
Impact of Elp3 on *B. bassiana* virulence and virulence-related properties. (**A**,**B**) Insect bioassay survival curves and calculated LT_50_ values using the greater wax moth (*G. mellonella*) larvae as hosts after topical application (immersion) and intra-hemocoel injection, respectively. (**C**) Representative images of fungal outgrowth on the surfaces of cadavers 4 d post death. (**D**,**E**) Quantification of fungal biomass, total extracellular protease (ECEs), and Pr1 protease activities after 3 d growth in CZB-BSA. (**F**) Representative microscopic images of submerged hyphae and blastospores after 3 d growth in CZB and TPB. Scale bars = 20 μm. (**G**,**H**) Fungal biomass levels and blastospore yields quantified after 3 d growth in CZB and TPB. Asterisk indicates significant difference (*p* < 0.05) from unmarked (Tukey’s HSD). Error bars: SD from three technical replicates.

To examine the contribution of *BbElp3* in *B. bassiana* pathogenicity, insect bioassays using *G. mellonella* larvae as the host were conducted via both topical application and intra-hemocoel injection (Figure 4A,B). In topical bioassays, the median lethal time to kill 50% of the target hosts (LT_50_) for the wild-type strain and complemented strains were 4.23 ± 0.22 d, whereas for the Δ*BbElp3* strain, the LT_50_ = 5.40 ± 0.14 d (~30% increase, i.e., decreased virulence, *p* < 0.001). For intra-hemocoel injection assays, the wild-type and complemented strain LT_50_ values were = 4.27 ± 0.15 d, while for the Δ*BbElp3* strain, the LT_50_ = 5.35 ± 0.28 d (~25% increase, *p* < 0.001). Moreover, after 4 d post death, fungal outgrowth on cadavers for the Δ*BbElp3* strain was noticeably less than that seen for the control strains (Figure 4C). In addition, the total enzymic activity of extracellular proteases and subtilisin-like proteases produced by *B. bassiana* that contribute to host cuticle degradation were examined. When cultivated on minimal CZB media, total fungal dry mass for the Δ*BbElp3* strain was significantly lower (~30% decrease, *p* < 0.001) than the control strains (Figure 4D). However, even after normalization to this difference in growth, the Δ*BbElp3* strain showed decreased activity towards the degradation of the protease substrate azo-casein (~60%, *p* < 0.001, total extracellular protease activity) as well as decreased Pr1 protease activity (75%, *p* < 0.001) (Figure 4E).

The attenuated intra-hemocoel virulence suggested that the transition of penetrating hyphae to the production of the in vivo blastospores (also termed hyphal bodies) that occurs in the insect hemocoel was impaired in the Δ*BbElp3* mutant. To test this hypothesis, fungal cells of each strain were collected after 3 d of growth in CZB or TPB (trehalose peptone broth that mimics the insect hemolymph), and the production of unicellular blastospores was quantified (Figure 4F). Compared to the wild-type and complemented mutants, the Δ*BbElp3* mutant showed significantly decreased total biomass production in the CZB (~30%, *p* < 0.001) and in the TPB media (~27%, *p* < 0.001) (Figure 4G). In terms of blastospore production, consistent with the microscopic visualization, the Δ*BbElp3* mutant showed ~25% decreased blastospore yield (*p* < 0.01) in both CZB and TPB cultures compared to the wild type (Figure 4H).

### 3.6. Global Regulatory Role of BbElp3 in Gene Transcription

**Figure 5:**
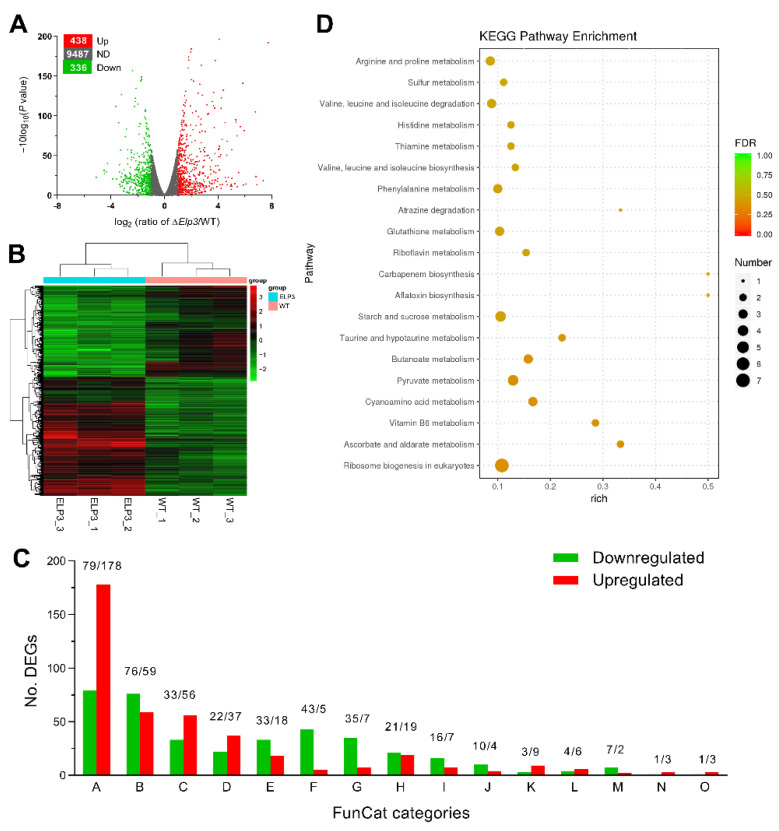
Comparative transcriptomic analyses of Δ*BbElp3* and wild-type *B. bassiana*. (**A**) transcriptome. (**A**) Distributions of *p*-values and ratios for the genes identified to be downregulated, upregulated, or not differentially regulated (ND) in Δ*BbElp3* versus the wild type. (**B**) Cluster analysis of the 775 differentially expressed genes (DEGs) found in the transcriptomic analyses of the Δ*BbElp3* mutant versus wild-type strains. (**C**) FunCat annotation into 15 functional categories of significantly regulated genes in Δ*BbElp3* versus wild type. A, metabolism; B, protein with binding function or cofactor requirement; C, cell rescue, defense, and virulence; D, cellular transportation; E, protein fate; F, transcription; G, cell cycle and DNA processing; H, biogenesis of cellular components; I, cellular communication/signal transduction mechanism; J, regulation of metabolism and protein function; K, energy; L, cell type differentiation; M, protein synthesis; N, systemic interaction with the environment; O, cell fate. (**D**) Counts and confidence intervals of differentially expressed genes enriched into the top 20 KEGG pathways in Δ*BbElp3* versus the wild type.

To examine the global gene networks affected by loss of *BbElp3*, a comparative transcriptomic analysis was performed as detailed in the Methods section. A total of 775 differentially expressed genes (DEGs) were identified between the Δ*BbElp3* mutant and *B. bassiana* wild-type cells (Tables S2 and S3). Among them, 336 (3.24% of the annotated genome) DEGs were downregulated [Log_2_ (ratio): −5.09 to −1.00], while 438 (4.23% of genome) were upregulated [Log_2_ (ratio): +1.00 to +7.73] in the Δ*BbElp3* mutant as compared to the wild type (Figure 5A,B). Approximately 47% of the total DEGs were classified into 15 functional classes via FunCat category annotation (Figure 5C). Among the classified DEGs, 69% were involved in metabolism, followed by 37% in binding or cofactor requirement; 24% in cell rescue, defense, and virulence; 16% in cellular transportation; 14% in protein fate; 13% in transcription; 11% in cell cycle and DNA processing; 11% in biogenesis of cellular components; 6.3% in cellular communication/signal transduction mechanism; 3.8% in regulation of metabolism and protein function; 3.3% in energy; and <3% in cell type differentiation, protein synthesis, systemic interactions with the environment, and cell fate (Figure 5C).

Among those genes differentially expressed in Δ*BbElp3*, 62 DEGs (Table S4) were found to participate in DNA processing, transcription, and cell cycle control. Twelve downregulated genes were involved in DNA restriction, modification, and DNA repair, including two DEAD/DEAH box helicases, a tRNA (Uracil-5-)-methyltransferase, a UV-endonuclease, UvdE, and the DNA-repair protein RAD14. A cluster of forty downregulated genes were involved in RNA synthesis and rRNA processing, including six DEAD/DEAH box helicases, three U3 small nucleolar RNA associated protein, four rRNA processing proteins, four transcription factors, and several proteins related to ribosome biogenesis, whereas among the upregulated genes were four involved in RNA modification and transportation. An additional six upregulated genes were involved in chitin dynamics, with eighteen downregulated genes annotated to be involved in mitotic cell cycle control and cytokinesis, including three protein kinases, the nuclear GTP-binding protein NUG1, one cyclin protein, and the SDA1 protein required for normal organization of the actin cytoskeleton.

A total of 58 DEGs in Δ*BbElp3* were found to be involved in cellular transport (Table S5). In terms of electron and (non-) vesicular transport and substrate transformations, thirty-six DEGs were upregulated, including nine cytochrome P450s, seven different kinds of transporters, and six dehydrogenase and four oxidases participating in the electron respiratory chain pathway. However, a set of twenty-two DEGs were downregulated, including nine different kinds of transporters, two oxidases, and five amino acid permeases, with the latter mainly involved in the urea cycle. A total of twenty-eight DEGs were identified to be involved in cellular communication, cell fate, and cell type differentiation (Table S6). Sixteen DEGs were downregulated including six protein kinases (protein kinase, histidine/serine kinase, CAMK kinase, casein kinase), one protein phosphatase (protein phosphatase 2C), and a Rho-related GTP-binding protein RhoE involved in G-protein signaling and hyphal septation regulation [47], whereas twelve upregulated genes included one aspartokinase, one annexin, and two catalytic enzymes involved in ATP synthesis.

A set of 89 DEGs in Δ*BbElp3* found to be involved in cell rescue, defense, and virulence (Table S7). In terms of the multi-stress tolerance, fourteen genes were upregulated in the Δ*BbElp3* mutant, including two ABC transporters, a ATPase protein, and a heat-shock protein Hsp70, while thirteen genes were downregulated, including two proline dehydrogenases, a sugar 1,4-lactone oxidase, a P-type ATPase family protein, an rRNA endonuclease NOB1, and the DNA-repair protein RAD14 involved in DNA damage repair process. With respect to annotation related to virulence and defense, forty-six genes were found in the upregulated dataset, including thirteen cytochrome P450s, four major facilitator superfamily (MFS) transporters, two ABC multidrug transporters, and several hydrolases/substrate degradation enzymes involved in cellular defense and/or infection. Within the downregulated DEG dataset, twenty-four genes were identified, including three MFS transporters, two ABC multidrug transporters, and a number of cellular defense/virulence factors, including an aminotriazole resistance protein, an arrestin domain-containing protein, carboxylesterase/peptidase, pectin lyase fold-virulence factor, and a subtilisin-like serine protease.

Analysis using the pathogen–host interaction (PHI) database revealed 80 DEGs (Table S8) in the dataset. Some of these DEGs are known to contribute significantly to virulence in *B. bassiana* [37]. Apart from 33 DEGs assigned the unaffected pathogenicity or increased virulence category, there were 29 upregulated genes involved in loss of pathogenicity or reduced virulence, including 4 cytochrome P450s, 2 ABC multidrug transporters, 2 C6 zinc finger domain-containing proteins (transcription factors), and several hydrolases/substrate degradation enzymes involved in fungal infection. An additional eighteen genes were downregulated, including two P-type ATPases, two phosphoesterase-like proteins, and a hydrophobin protein involved in fungal adherence to the host cuticle.

Using KEGG classification analyses, 64 DEGs in Δ*BbElp3* were significantly enriched into 57 different (KEGG) pathways (detailed in Table S9). Among the top 20 enriched KEGG pathways (Figure 5D), many DEGs were involved in translation (ribosome biogenesis), carbohydrate metabolism (e.g., ascorbate and aldarate, pyruvate, butanoate, starch, and sucrose metabolism), and amino acid metabolism (including cyano-amino acid, taurine and hypotaurine, glutathione, phenylalanine, histidine, valine, leucine, and isoleucine metabolism as well as in valine, leucine, and isoleucine biosynthesis). A set of DEGs were also found functionally clustered in processes involving the metabolism of cofactors and vitamins, including vitamin B6, riboflavin, and thiamine metabolism, as well as in the biosynthesis of secondary metabolites (e.g., gene belonging to an aflatoxin-like cluster and carbapenem biosynthesis).

Among the top 20 downregulated genes identified in the Δ*BbElp3* versus the wild type using these analyses (Table S10), several genes were found to participate in iron transport and mitochondrial respiratory chain functioning. These included a sodium symporter, an ATPase protein, and a FAD-dependent oxidoreductase. Similar to other analyses described above, the DEG dataset analyzed via KEGG also found genes involved in fungal multi-stress tolerance (e.g., ABC transporter, UV-endonuclease UvdE, and phosphatase 2C) and metabolism (e.g., salicylate hydroxylase, longiborneol synthase, and a trypsin-related protease). Within the top 20 upregulated genes, three hydrolases/degradation enzymes, including two alpha/beta hydrolases, and an arginosuccinate lyase were identified. Moreover, two LysM-domain-containing proteins implicated in fungal pathogenicity [48] were upregulated in Δ*BbElp3*. Interestingly, two methyltransferases and a SCF E3 ubiquitin ligase protein were also differentially regulated in Δ*BbElp3*, indicating possible cross-talk between methylation/ubiquitination and the acetylation mediated by BbElp3. Within the top differentially expressed genes, essentially half, i.e., nine downregulated genes and ten upregulated genes, were uncharacterized/unknown genes.

## 4. Discussion

Chromatin remodeling involving histone protein acetylation/deacetylation affects global genes, including those specialized for successful fungal infection [49]. Such epigenetic marks are dynamic and follow sequential and spatial patterns along the histones that organize cellular DNA to affect transcriptional programs that can be inherited by daughter cells. In fungi, HATs and HDACs have been shown to have important roles in affecting virulence in plant, insect, and animal (human) pathogens [50,51,52,53,54]. As a saprophyte, facultative plant mutualist, and facultative (insect) pathogen, *B. bassiana* has adapted to radically different environmental niches that likely require activation of different cellular pathways for successful survival in these contrasting environments, including conditions of osmotic, oxidative, thermal, UV, pH, and hypoxic stress as well as competition with other microbes [55,56,57,58]. Thus far, in *B. bassiana*, the contributions of several HATs and HDACs have been investigated, and each appears to control distinct (though sometimes overlapping) sets of gene networks, subsequently impacting different (though, again, sometimes overlapping) phenotypes. Previous studies have examined four *B. bassiana* HATs, including the GNAT superfamily members Gcn5 and Spt10, the MYST superfamily member Mst2, and the P300/CBP family member Rtt109, as well as four HDACs, including a Class I HDAC (Rpd3), a Class II HDAC (Hos2), and two fungal sirtuins (Sir2 and SirT2) [38,39,40,41,44,59,60,61]. Here, we have extended these analyses to Elp3, a GNAT superfamily HAT, which has been shown to have pleiotropic roles in transcriptional activation and environmental adaptions in organisms ranging from yeast to humans [17,20,21,22,23] but has remained largely unexplored in filamentous fungi and uncharacterized in insect pathogenic fungi.

Our data show that the *B. bassiana* Elp3 homolog is localized to mitochondria (with cellular and nuclear localization also noted), similar to what has been reported in the (vertebrate) parasitic protozoan *Toxoplasma gondii* as well as in mammalian systems [22,23] but apparently different from what has been reported for Elp3 in the rice blast fungus, where nuclear localization was found, although in the latter case, it is unclear whether the authors also looked for mitochondrial localization [25]. Interestingly, in the Δ*BbElp3* transcriptome, an array of mitochondrial proteins were found differentially regulated, including two mitochondrial chaperones and three mitochondrial carrier proteins as well as eleven catalytical/binding proteins functioning in respiratory chains, including seven ATPase proteins, two ATP synthethases, and two ATP-binding proteins, indicating potential roles of BbElp3 in mitochondrial function and energy utilization. This raises the intriguing possibility that Elp3 has mitochondrial (or other) protein targets distinct from its functioning in histone acetylation. This may be more common than previously thought since it has been shown that at least one *B. bassiana* HDAC, namely BbRpd3, also has targets beyond canonical histones [59]. Consistent with the fact that Elp3 does function as a HAT, Western blotting analyses revealed that loss of *BbElp3* significantly (by 50%) reduced bulk acetylation of histone H3, similar to results seen in *S. cerevisiae* and *Homo sapiens* (Primates, Mammalia) [9,10]. These results imply that at least sufficient levels of Elp3 are found in the nucleus to mediate this activity. In yeast, Elp3 has been suggested to have broad effects in governing gene transcription, with potential overlap in function with the SAGA histone acetyltransferase complex [17,62]. As expected, due to its effects on chromatin remodeling, transcriptomic analyses revealed changes in the expression of a large number of genes (775) in Δ*BbElp3* cells as compared to its wild-type parent, and our data indicate a more severe overall growth defect phenotype as compared to what has been reported in yeast [9,17]. Curiously, despite having “activating” functioning (which would suggest that loss of its activity would have a dampening effect on global gene transcription), a greater number of upregulated than downregulated genes were identified in the *BbElp3* mutant strain, suggesting a suppressor role for BbElp3, i.e., that important targets of BbElp3 transcriptional activation may be repressors.

Conidia are typically the active ingredient used in insect biological control applications using *B. bassiana*; thus, conidial capacity and activity are crucial parameters for successful pest control applications [35]. In addition, conidial viability, spore dispersal, and the conditions under which conidia are grown are known to affect virulence and/or the biological control potential of the fungus [63,64,65] In the Δ*BbElp3* mutant, conidial yield was significantly reduced, likely due to the G1 phase arrest seen for Δ*BbElp3* cells, as an ordered cell cycle is known to be critical for asexual growth and spore production, and the ability of the fungus to grow on various carbon and nitrogen sources was reduced [66,67]. The delayed cell cycle progression is also consistent with the observed enlarged conidia/blastospores and the longer hyphal cell growth with reduced septation. Correspondingly, the RNA-Seq analysis of the Δ*BbElp3* mutant revealed misregulation of a large array genes involved in DNA processing, transcription, and cell cycle control, including DNA helicases, RNA processing proteins, protein kinases, and cyclins. Moreover, the expression of *RhoE*, a Rho family G protein critical for septation and cytokinesis [47], was significantly decreased in the Δ*BbElp3* mutant, which may help account for the disrupted hyphal septation pattern seen in the mutant.

The ability of conidia to tolerate various stress and complicated environments is also critical for the biological potential of fungal strains [35,68,69,70]. In our study, loss of *BbElp3* resulted in decreased conidial tolerances to nearly all kinds of stress conditions tested, including osmotic stress, oxidative stress, heat, and antibiotic stress. Potentially accounting for aspects of these phenotypes, a large array of cellular transporter genes were found to be significantly downregulated in the Δ*BbElp3* mutant, including six MFS transporters, three ABC transporters, and two OPT oligopeptide transporters. Five (P-type) ATPases, which have been shown to be important for electron transport chain functioning [71], were also significantly downregulated in the Δ*BbElp3* mutant. The high-osmolarity glycerol (HOG) MAPK pathway is known to function in cell tolerance to osmotic stress in *B. bassiana* [72], and expression of the HOG pathway histidine kinase *Sln1* was found to be significantly downregulated in the mutant. Expression of the heat-shock protein Hsp20 family chaperone was also significantly decreased, likely helping to account for the significant heat-sensitive phenotype of the Δ*BbElp3* mutant.

In addition, the loss of *BbElp3* attenuated the ability of *B bassiana* to infect insect hosts via topical application, which represents the natural route of infection in which conidia must first adhere and then penetrate the host cuticle. Virulence was also reduced in intrahemocoel-injection assays, where conidia bypass the need to penetrate the cuticle but are directly in contact with the host (innate) immune systems. These results indicate impairment of both penetration and immune evasion processes involved in successful infection as a result of loss of Elp3 activity. Within this context, our data indicated a significant reduction in the production of critical cuticle degrading enzymes, including the proteases and subtilisin-like proteases [73], consistent with the impaired topical infection ability of the Δ*BbElp3* mutant. Further consistent with the bioassay results, the transcriptomics analyses indicated the downregulation of a large array of genes involved in cuticle penetration and hemocoel infection in Δ*BbElp3* mutant as compared to the wild type, e.g., two subtilisin-like proteases vital for cuticle degradation and invasion [73]; hydrophobins, important for fungal conidial adhesion to the host cuticle [74]; a lysine motif (LysM) effector, important for chitin-induced immunity and fungal virulence in *B. bassiana* [48]; and genes involved in oosporein synthesis, a secondary metabolite implicated in successful outgrowth on cadavers [34]. Overall, BbElp3 likely exerts its role in affecting virulence through: (1) disturbing cell cycle progression, (2) impairing conidial tolerances to various stress conditions (both oxidative and osmotic stress occur during the infection process), and (3) via transcriptional regulating of a set of virulence-related genes. It should be noted that a number of important virulence-related gene pathways were found to be upregulated in the Δ*BbElp3* mutant as compared to the wild type, suggesting some compensatory effects that may account for the continued, albeit lower, virulence seen for the mutant. In conclusion, our data have identified a range of biological contributions of BbElp3 in *B. bassiana* asexual development, cell cycle control, multi-stress tolerance, and carbon/nitrogen metabolism as well as towards the infection process.

## 5. Conclusions

In the *B. bassiana*-insect host–pathogen system, our data show that the Elongator subunit Elp3: (i) mediates histone acetyltransferase activity on histone H3; (ii) is mainly localized to mitochondria, with nuclear and cytoplasmic localization also seen; and (iii) contributes to wide range of cellular processes, including stress response, virulence, and autophagy. Consistent with the pleiotropic phenotypes observed, loss of *BbElp3* results in altered regulation of a large array of downstream genes involved in a wide range of biological processes, including DNA processing, asexual development, cell cycle, stress response, and virulence. Intriguingly, in Δ*BbElp3*-specific transcriptome, 252 DEGs could not be classified, i.e., were to uncharacterized proteins, indicating significant room for further investigation. Despite the large range of targets, directed regulation of protein kinases, hydrophobins, multidrug transporters, and other pathogenicity-related proteins at the transcription levels suggest discrete pathway(s) by which Elp3 controls gene transcription, cell cycle, stress resistance, and virulence. The mitochondrial localization also suggests that investigations seeking to identify non-histone targets of Elp3 are warranted. Our data revealed a broad regulating role of Elongator protein HAT Elp3 in transcription of a large array genes and biological potential in *B. bassiana*.

## Data Availability

All transcriptomics data have been deposited to the Sequence Read Archive (SRA) on NCBI (https://www.ncbi.nlm.nih.gov/, accessed on 13 April 2021) with the dataset identifiers PRJNA721731.

## Supplementary Materials

The following supporting information can be downloaded at: https://www.mdpi.com/article/10.3390/jof8080834/s1, Figure S1: Bioinformatic analysis of fungal Elp3 homologues; Figure S2: Construction and identification of *B. bassiana Elp3* mutants; Figure S3: Impact of *BbElp3* deletion on carbon/nitrogen metabolism; Table S1: Primers used for genetic manipulation of *Elp3* in *B. bassiana*; Table S2: Lists of 775 differentially expressed genes in Δ*Bb**Elp3* versus WT; Table S3: The RPKMs of the 775 DEGs in the heatmap of Δ*BbElp3* versus WT; Table S4: Lists of 62 Elp3-regulated genes involved in DNA events, cell cycle, and transcription of *B. bassiana*; Table S5: Lists of 58 Elp3-regulated genes involved in cellular transportation of *B. bassiana*; Table S6: Lists of 28 Elp3-regulated genes involved in cell communication and morphogenesis of *B. bassiana*; Table S7: Lists of 89 Elp3-regulated genes involved in cell rescue, defense, and virulence of *B. bassiana*; Table S8: Lists of 80 BbElp3-regulated genes enriched in pathogen-host interaction (PHI) networks; Table S9: Lists of 64 DEGs enriched in 57 KEGG pathways in Δ*BbElp3* versus WT; Table S10: Lists of the top 20 upregulated genes and the top 20 downregulated genes in Δ*BbElp3* versus WT.
