# Supplementary material for: The Elongator Subunit Elp3 Regulates Development, Stress Tolerance, Cell Cycle, and Virulence in the Entomopathogenic Fungus Beauveria bassiana
Source: J Fungi (Basel). 2022 Aug 10;8(8):834. doi: 10.3390/jof8080834 (PMC9410351; doi:10.3390/jof8080834)
Supplement: Supplementary file 1 [file jof-08-00834-s001.zip › jof-1854723-supplementary.pdf]

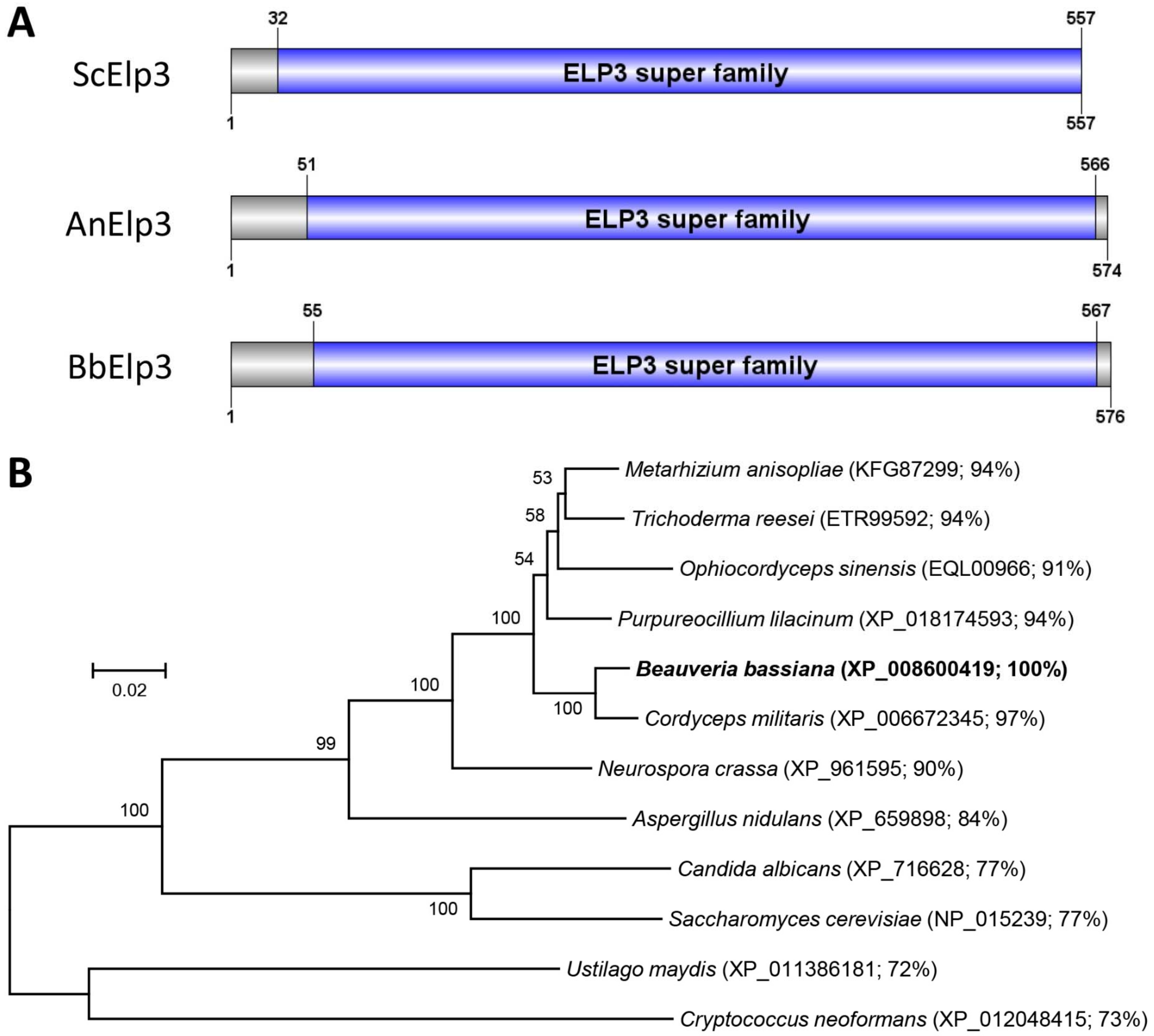

Figure S1. Bioinformatic analysis of fungal Elp3 homologues. (**A**) Sequence features of Elp3 homologs found in *Beauveria bassiana* (Bb), *Saccharomyces cerevisiae* (Sc), and *Aspergillus nidulans* (An). The domain of each protein was predicted at https://blast.ncbi.nlm.nih.gov/Blast.cgi. (**B**) Phylogenetic relationship of *B. bassiana* Elp3 with the homologs found in other representative fungi. A neighbor-joining method in MEGA7 at http://www.megasoftware.net was used in the phylogenetic analysis. Each fungal name is followed by the NCBI accession code of each protein and its sequence identity (%) to *B. bassiana* Elp3 in parentheses. Poisson model was used with 1000 bootstrap replications in uniform rates. Scale bar: branch length proportional to genetic distance assessed with the neighbor-joining method.

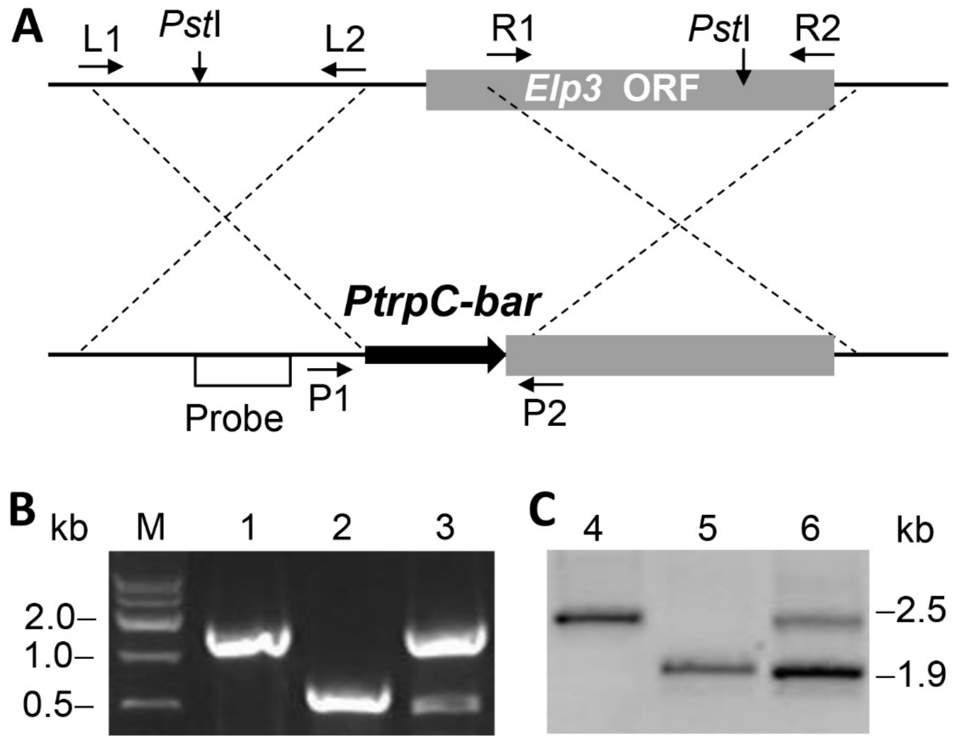

Figure S2 Construction and identification of *B. bassiana Elp3* mutants. (**A**) Schematic diagram for the strategy of *Elp3* deletion. (**B**) The *Elp3* mutants identified via PCR (lanes 1–3) and Southern blotting (lanes 4–6) analyses with paired primers and amplified probe (Table S1). Lanes 1 and 4: *ΔElp3* mutant. Lanes 2 and 5: wild-type. Lanes 3 and 6: *ΔElp3::Elp3* mutant. Genomic DNAs were digested with *Pst*I at the marked sites for the Southern blotting hybridization of *Elp3*.

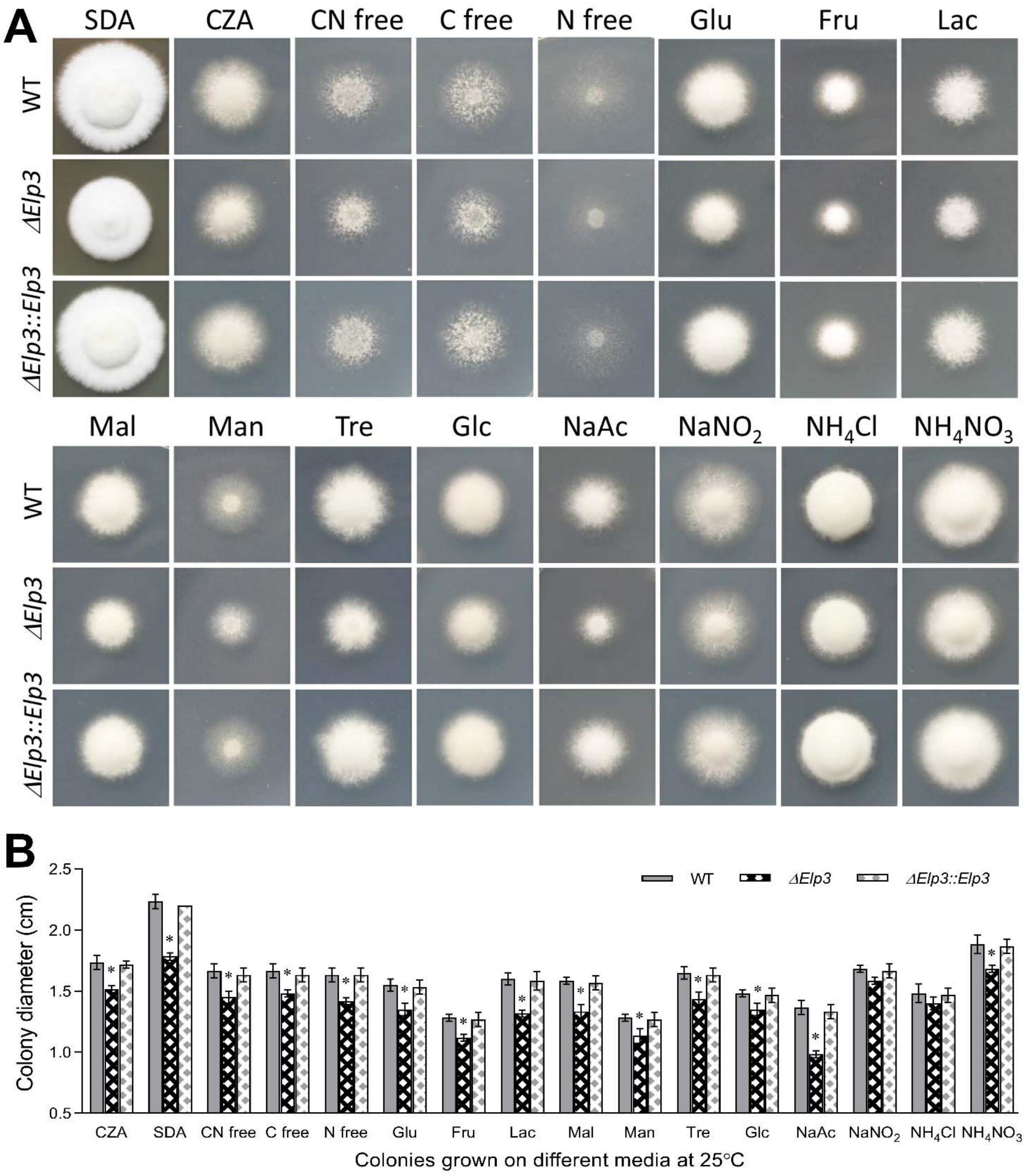

Figure S3 Impact of *BbElp3* deletion on carbon/nitrogen metabolism. **(A,B)** Representative images and colony diameters after 8 d inoculation at 25ºC on tested media. Carbon sources tested: Glu; glucose, Fru; fructose, Lac; lactose, Mal; maltose, Man; mannitol, Tre; trehalose, Glc; glycerol, NaAc. Nitrogen sources tested: $NaNO_2$, NH_4Cl or NH_4NO_3. Additional media: carbon-deleted (C free), nitrogen-deleted (N free) or both deleted (CN free). All colonies were initiated by spotting 1 µl of 1×10^6 conidia/ml suspension on the plates. All experiments were performed three times. Error bars = ± SD. Asterisks indicate significantly different from unmarked (Tukey's HSD, * indicates $p < 0.05$).

Table S1. Primers used for genetic manipulation of *Elp3* in *B. bassiana*.

Primers	Paired sequences (5'-3')*	Purpose
cElp3-F/R	CCCGGGACTAGTGATATCATGGCTACCGCTACAGTCA / CTTGCTCACCATGAATTCTTCTTCAGCCGGCCCT	Cloning *Elp3* cDNA(1728 bp)
Elp3up-F/R	AAAAACCCGGGCTGCTCTCTCCAGCCTCATTC / AAAAAAAGCTTTCTTCTGCAATGACCGTCTGT	Cloning *Elp3* 5′-end (1618 bp)
Elp3dn-F/R	AAAAAAGATCTGCGACATCAACCTCAACAGC / AAAAAACTAGTGCCAACCCCGATTCTATTTT	Cloning *Elp3* 3′-end (1676 bp)
Elp3fl-F/R	ggggACCACTTTGTACAAGAAAGCTTGCGTCCGGTATGTGGTGA / ggggACAAGTTTGTACAAAAAAGCAACCGAGCCGTGGTAAATGG	Cloning full-length *Elp3* (4416 bp)
pElp3-F/R	CTGCCCCACCTTGACACATC / TGTAGTGTTCGGGGATAGCG	PCR detecting *Elp3*
sbElp3-F/R	CTTGTCTTGATGTGACCTCGTA/ GCTGGTAATTGCCCGTAT	Southern blotting *Elp3* (420 bp)

* Underlined regions denote the restriction enzyme sites used for cloning *Elp3* cDNA (*Spe*I/*EcoR*I) or deleting *Elp3* (*Xma*I/*Hind*III and *Bgl*II/*Spe*I) and the fragments of gateway exchange for *Elp3* complementation.
